# Specific CpG hyper-methylation leads to *Ankrd26* gene down-regulation in white adipose tissue of a mouse model of diet-induced obesity

**DOI:** 10.1038/srep43526

**Published:** 2017-03-07

**Authors:** Gregory A. Raciti, Rosa Spinelli, Antonella Desiderio, Michele Longo, Luca Parrillo, Cecilia Nigro, Vittoria D’Esposito, Paola Mirra, Francesca Fiory, Vincenzo Pilone, Pietro Forestieri, Pietro Formisano, Ira Pastan, Claudia Miele, Francesco Beguinot

**Affiliations:** 1 URT of the Institute of Experimental Endocrinology and Oncology “G. Salvatore”, National Council of Research, Naples, 80131, Italy; 2 Department of Translational Medical Sciences, University of Naples “Federico II”, Naples, 80131, Italy; 3 Bariatric and Metabolic Surgery Unit, University of Salerno, Salerno, 84084, Italy; 4 Department of Clinical Medicine and Surgery, University of Naples “Federico II”, Naples, 80131, Italy; 5 Laboratory of Molecular Biology (LMB), National Cancer Institute (NCI), National Institute of Health (NIH), Bethesda, MD 20892, USA

## Abstract

Epigenetic modifications alter transcriptional activity and contribute to the effects of environment on the individual risk of obesity and Type 2 Diabetes (T2D). Here, we have estimated the *in vivo* effect of a fat-enriched diet (HFD) on the expression and the epigenetic regulation of the *Ankyrin repeat domain 26 (Ankrd26*) gene, which is associated with the onset of these disorders. In visceral adipose tissue (VAT), HFD exposure determined a specific hyper-methylation of *Ankrd26* promoter at the −436 and −431 bp CpG sites (CpGs) and impaired its expression. Methylation of these 2 CpGs impaired binding of the histone acetyltransferase/transcriptional coactivator p300 to this same region, causing hypo-acetylation of histone H4 at the *Ankrd26* promoter and loss of binding of RNA Pol II at the *Ankrd26* Transcription Start Site (TSS). In addition, HFD increased binding of DNA methyl-transferases (DNMTs) 3a and 3b and methyl-CpG-binding domain protein 2 (MBD2) to the *Ankrd26* promoter. More importantly, *Ankrd26* down-regulation enhanced secretion of pro-inflammatory mediators by 3T3-L1 adipocytes as well as in human sera. Thus, in mice, the exposure to HFD induces epigenetic silencing of the *Ankrd26* gene, which contributes to the adipose tissue inflammatory secretion profile induced by high-fat regimens.

Obesity and T2D are two common non-communicable diseases, which are now reaching epidemic proportions globally^1^,^2^,^3^. Epigenetic processes may contribute to the development of these disorders and mediate the effects of environmental exposure on risk of both diseases. Indeed, studies in humans and animal models support the association between changes in the nutritional status, epigenetic modifications and predisposition to obesity and T2D^4^,^5^,^6^,^7^.

White adipose tissue (WAT) is a major endocrine tissue actively involved in the maintenance of the metabolic homeostasis in response to nutrition and other environmental clues through changes in fat storage, tissue expansion and adipokine secretion^8^. In disease states, the failure of compensatory response results in an impaired endocrine function which leads to insulin resistance and metabolic derangement^9^. In particular, fat stored in VAT strongly correlates with metabolic alterations and has been shown to be an independent risk factor for obesity-associated comorbidities^10^,^11^,^12^.

*Ankrd26* was recently identified as a gene involved in the regulation of the feeding behavior and in the development of both obesity and T2D in mice^13^,^14^,^15^. *ANKRD26* maps at chromosome 10p12 in humans, a region associated with certain forms of hereditary obesity^16^. In mice, *Ankrd26* gene is highly expressed in both the hypothalamus and WAT and its partial inactivation induces marked hyperphagia, severe obesity and diabetes *in vivo*^13^,^14^. In addition, *in vitro* evidence indicates *Ankrd26* as a regulator of adipogenesis^17^,^18^. A methylome analysis of mouse epididymal WAT (eAT)^19^, the largest and easy accessible VAT depots in rodents^20^,^21^, has identified promoter hyper-methylation of *Ankrd26* gene in HFD-fed compared to age- and sex-matched chow diet-fed mice, suggesting that *Ankrd26* gene is amenable to nutritionally-induced epigenetic modifications. In this study, we aimed at establishing whether and how HFD modulates *Ankrd26* gene expression in VAT *in vivo* through epigenetic processes.

## Results

### HFD affects body weight, glucose homeostasis and insulin sensitivity in mice

HFD-fed mice were heavier than standard chow diet (STD)-fed mice and reached a 50% increase of body weight compared with controls after 22 weeks of diet regimens (Table 1). These mice also exhibited increased fasting blood glucose levels, impaired glucose tolerance upon glucose loading and reduced insulin sensitivity after insulin injection compared with control mice (Supplementary Fig. S1).

### HFD impairs *Ankrd26* expression in mice

**Figure 1:**
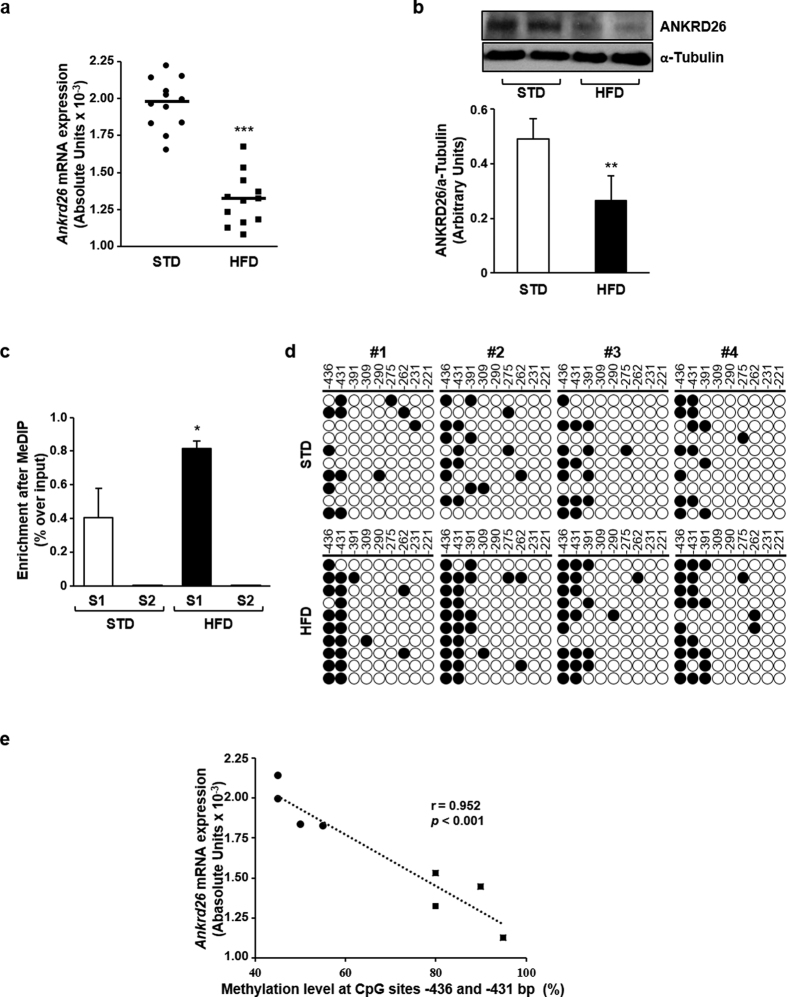
Ankrd26 expression and DNA methylation in eAT from mice upon 22 weeks of HFD or STD treatments. (**a**) qPCR of *Ankrd26* mRNA for HFD- (n = 12) and STD-fed (n = 12) mice. mRNA levels are expressed in absolute units (AU). (**b**) Representative western blot for ANKRD26 and α-Tubulin. Uncut western blot images are in the Supplementary Fig. S5. (**c**) MeDIP-qPCR of segment 1 (S1; −462 bp/−193 bp) and segment 2 (S2; −158 bp/+140 bp) of *Ankrd26* promoter region. (**d**) Bisulfite sequencing of *Ankrd26* promoter region (−436 bp/−221 bp) in HFD- (n = 4) and STD-fed (n = 4) mice. Each row indicates sequencing results of ten independent clones. White circles, un-methylated CpGs; black circles, methylated CpGs. CpG position relative to *Ankrd26* TSS is shown above each column. (**e**) Correlation between DNA methylation percentage at CpGs −436 and −431 bp and *Ankrd26* mRNA levels. *r* and *p* values are indicated on graph. (**b**,**c**) Results are mean ± SD from three independent experiments. (**a**–**c**), *p < 0.05, **p < 0.01, and ***p < 0.001 *vs* STD.

To establish whether HFD modulates *in vivo Ankrd26* expression, mRNA and protein levels were measured in eAT. Treatment with HFD for 22 weeks led to a significant decrease in both *Ankrd26* mRNA (*p* < 0.001) and protein (*p* < 0.01) levels in obese mice compared with controls (Fig. 1a and b). Similarly, HFD lowered *Ankrd26* mRNA levels in mesenteric VAT (Supplementary Fig. S2). HFD treatment for 4 additional weeks did not elicit any further decrease in *Ankrd26* mRNA expression in the HFD-fed mice (34 week-old STD, *Ankrd26* mRNA: 2.29 × 10^−3^ ± 0.11 × 10^−3^ AU; 34 week-old HFD, *Ankrd26* mRNA: 1.36 × 10^−3^ ± 0.19 × 10^−3^ AU; *p* < 0.001). Differently from the long-term treatment, both *Ankrd26* mRNA and protein levels showed no differences between HFD- and STD-fed mice upon 8 weeks of diet regimens (Supplementary Fig. S3a and b). Next, the *Ankrd26* gene expression was assessed *in vitro* by exposing 3T3-L1 adipocytes to either palmitate or oleate, representing saturated and unsaturated fatty acid species, which are abundant in the HFD, or alternatively to leptin, whose levels raise through obesity development^22^. Quantitative real-time PCR (qPCR) analysis showed that palmitate, but not oleate or leptin, reduced *Ankrd26* expression by about 25% (Supplementary Fig. S4a and b), suggesting that, at least in part, excess of saturated fats accounts for HFD-induced *Ankrd26* gene repression.

### HFD induces DNA methylation at the *Ankrd26* promoter in mice

To discover whether HFD induces DNA methylation changes at the *Ankrd26* promoter and 5′-untraslated region (5′ UTR), we performed Methylated DNA Immunoprecipitation (MeDIP) assay on pooled eAT genomic DNA from STD- and HFD-fed mice. This analysis revealed a 2-fold increase in DNA methylation at a segment of the promoter region (S1; −462 bp/−193 bp) in HFD-fed mice, while no DNA methylation enrichment was observed in a second segment (S2; −158 bp/ + 140 bp; Fig. 1c). Consistently, palmitate but not oleate or leptin, enhanced S1 DNA methylation at the *Ankrd26* promoter in 3T3-L1 adipocytes (Supplementary Fig. S4c and d), as showed by MeDIP assay. To further determine the specific HFD-induced DNA methylation profile occurring at 9 CpGs located at −436 and −221 bp from the *Ankrd26* TSS, we adopted bisulfite sequencing analysis. High CpG methylation density was detected in obese mice compared with controls in 2 close cytosine residues at −436 and −431 bp from the *Ankrd26* TSS (Fig. 1d). The combined percentage of methylation at these sites was inversely related to the amount of *Ankrd26* mRNA (Fig. 1e). In parallel with mRNA expression, mice fed HFD or STD for 8 weeks showed no difference in the *Ankrd26* DNA methylation state (Supplementary Fig. S3c). In addition, in 16 week-old and 30 week-old STD-fed mice, no difference in both *Ankrd26* mRNA levels (16 week-old STD, *Ankrd26* mRNA: 2.10 × 10^−3^ ± 0.20 × 10^−3^ AU; 30 week-old STD, *Ankrd26* mRNA: 1.96 × 10^−3^ ± 0.23 × 10^−3^ AU; *p* = 0.126) and DNA methylation (16 week-old STD, DNA methylation: 51.7 ± 2.9%; 30 week-old STD, DNA methylation: 51.3 ± 4.8%; *p* = 0.900) were observed. All together, these data indicate that the long-term exposure to calorie overload, rather than aging, affects eAT *Ankrd26* expression and DNA methylation in mice.

### Methylation at the CpGs −436 and −431 bp modulates *Ankrd26* promoter activity

**Figure 2:**
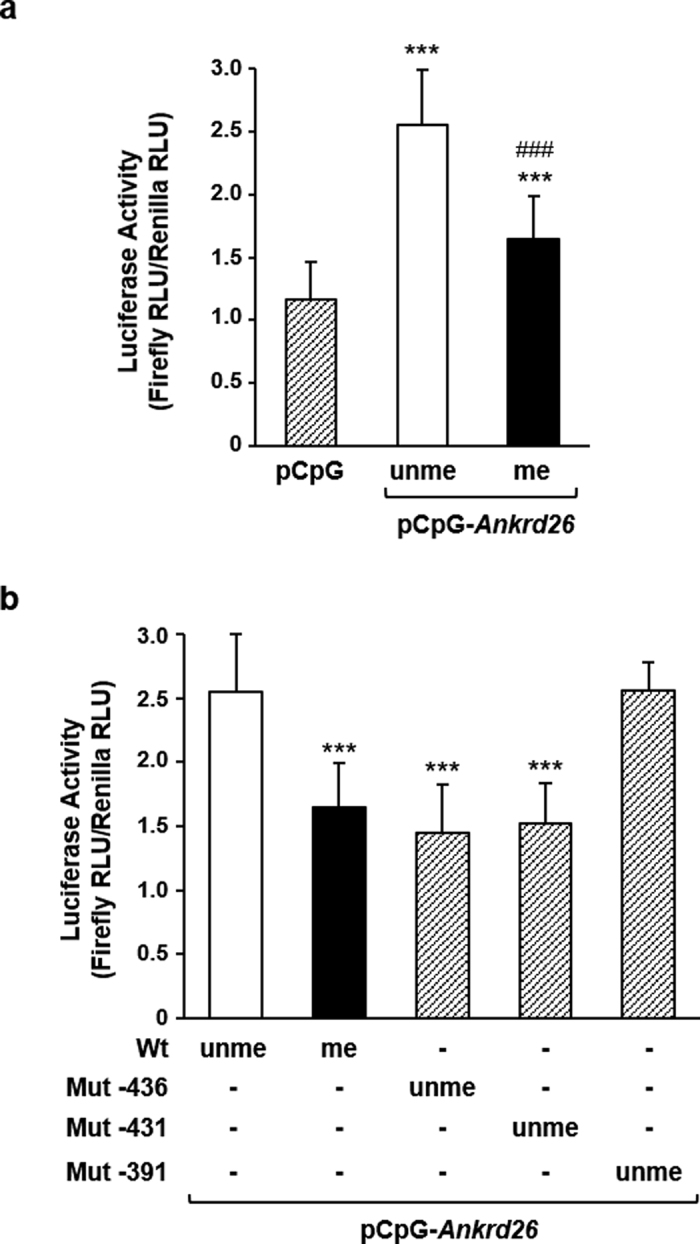
*Ankrd26* promoter activity in NIH-3T3 cells. (**a**) Luciferase activity of pCpG-*Ankrd26* constructs *in vitro* methylated (me) or un-methylated (unme) and of pCpG empty vector. Firefly luciferase activity was normalized to Renilla luciferase activity. Luciferase activity was measured in relative light units (RLU). ***p < 0.001 *vs* pCpG; ^###^*p* < 0.001 *vs* pCpG-*Ankrd26*unme. (**b**) Luciferase activity of unme mutagenized vectors, pCpG-*Ankrd26*-436, pCpG-*Ankrd26*-431 and pCpG-*Ankrd26*-391. Firefly luciferase activity was normalized to Renilla luciferase activity. Luciferase activity was measured in relative light units (RLU). ***p < 0.001 *vs* Wt unme. (**a**,**b**) results are mean ± SD from three independent experiments.

To evaluate the causal relationship between the promoter DNA methylation and transcription of *Ankrd26* gene, a luciferase assay was performed in NIH-3T3 cells transfected with *in vitro* methylated (me) or un-methylated (unme) pCpG-*Ankrd26* luciferase reporter vectors, in which a selected region of the *Ankrd26* promoter was cloned, containing the CpGs −436, −431 and −391 bp. The un-methylated *Ankrd26* promoter induced a 2.5-fold increase in the luciferase activity compared with the empty vector (Fig. 2a), indicating that this selected fragment is sufficient to mediate promoter activity. In addition, methylation of the *Ankrd26* promoter caused a 35% decrease of luciferase activity compared with the un-methylated *Ankrd26* promoter (Fig. 2a), indicating that methylation of this region has a negative impact on *Ankrd26* gene expression. Next, to define whether the CpGs −436 or −431 bp or both is/are responsible for the regulation of the *Ankrd26* promoter activity, luciferase assays were performed in NIH-3T3 cells transfected with un-methylated site-specific mutagenized vectors. The un-methylated *Ankrd26* promoter mutagenized at the −436 bp CpG site (*Ankrd26*−436unme), similarly to the wild type (Wt) methylated *Ankrd26* promoter, showed a 40% reduction of the luciferase activity compared with the un-methylated *Ankrd26* Wt fragment (Fig. 2b). Similar data were obtained when the *Ankrd26* promoter was mutagenized at the −431 bp CpG site (Fig. 2b). Conversely, when the *Ankrd26* promoter was mutagenized at the −391 bp CpG site, the luciferase activity of the un-methylated *Ankrd26*−391 promoter was comparable to the un-methylated Wt promoter (Fig. 2b). Thus, specific methylation at the −436 and −431 bp CpGs in the *Ankrd26* promoter modulates *Ankrd26* gene expression *in vitro*.

### Methylation at the CpGs −436 and −431 bp impairs p300 binding to the *Ankrd26* promoter

**Figure 3:**
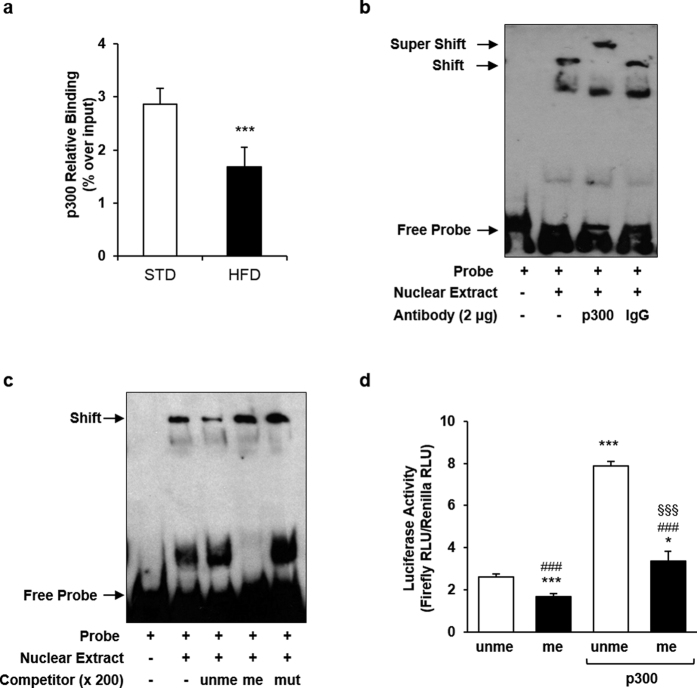
p300 binding and activity on *Ankrd26* promoter. (**a**) ChIP of p300 binding on *Ankrd26* promoter in eAT from HFD- (n = 3) and STD-fed (n = 3) mice, upon 22 weeks of diet regimens. ChIP enrichment is relative to Input chromatin. ***p < 0.001 *vs* STD. (**b**,**c**) Representative EMSA for double-stranded biotinylated *Ankrd26* probe with Nuclear Extract (NE) from NIH-3T3 cells. Uncut gel images are in the Supplementary Fig. S5. (**b**) EMSA super-shift assay with an anti-p300 antibody (lane 3) or a rabbit IgG (lane 4). (**c**) EMSA competition assay with 200-fold molar excess of un-labeled un-methylated (unme; lane 3), methylated (me; lane 4) or mutagenized (mut; lane 5) competitor. (**d**) Luciferase activity of *in vitro* methylated (me) or un-methylated (unme) pCpG-*Ankrd26* vector in NIH-3T3 cells co-transfected with pCl.*p300* vector. Firefly luciferase activity was normalized to Renilla luciferase activity. Luciferase activity was measured in relative light units (RLU). ***p < 0.001 *vs* pCpG-*Ankrd26* unme; ^###^*p* < 0.001 *vs* pCpG-*Ankrd26* unme + pCl.*p300*; ^§§§^*p* < 0.001 *vs* pCpG-*Ankrd26* me. (**a** and **d**), results are mean ± SD from three independent experiments.

DNA methylation often induces gene silencing by inhibiting transcriptional activator binding to promoters^23^. TFBIND analysis of the *Ankrd26* promoter region spanning the CpGs −436 and −431 bp, predicted a consensus sequence (−442 bp/−429 bp) for the histone acetyltransferase/transcriptional coactivator p300^24^,^25^. The recruitment of p300 to this putative binding site and its relevance to the regulation of the *Ankrd26* gene expression was therefore investigated. Chromatin Immunoprecipitation (ChIP) analysis showed a 40% decrease in p300 binding to the *Ankrd26* promoter in eAT of obese compared with lean mice (Fig. 3a). In addition, Electrophoretic Mobility Shift Assay (EMSA) with a double-stranded labeled probe containing the p300 consensus sequence on the *Ankrd26* promoter revealed that the addition of p300 antibody to the probe/Nuclear Extract (NE) mix super-shifted one of the complexes formed by interaction of the probe with the NE proteins (Fig. 3b). Also, the presence of an un-methylated competitor to the probe/NE mix effectively displaced p300 binding to the probe, while the probe/p300 complex was not affected by the addition of both a methylated or a mutagenized competitor (Fig. 3c). The *in vitro* over-expression of p300 in NIH-3T3 cells caused a 3-fold increase of the un-methylated *Ankrd26* promoter activity (Fig. 3d). At variance, when p300 was over-expressed, the luciferase activity of the methylated *Ankrd26* promoter was 60% lower compared with the un-methylated *Ankrd26* promoter (Fig. 3d). All together, these data indicate that p300 binding to *Ankrd26* promoter regulates *Ankrd26* gene expression and is dependent on the methylation state of the CpGs −436 and −431 bp.

### HFD induces hyper-methylation of the *Ankrd26* promoter through DNMT3a and DNMT3b in mice

**Figure 4:**
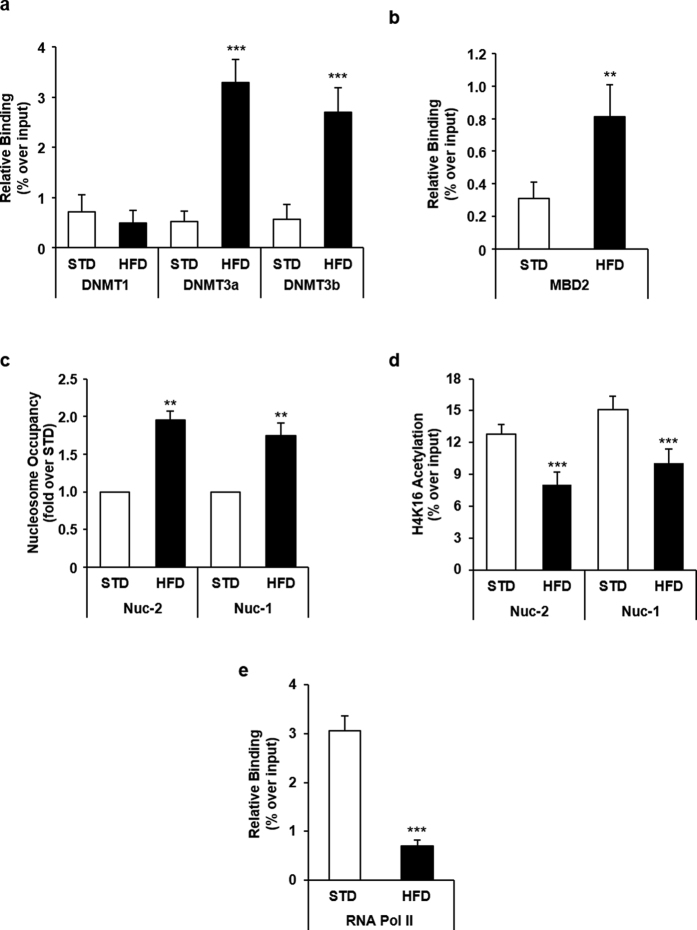
Epigenetic changes and protein binding at *Ankrd26* promoter in eAT from mice upon 22 weeks of HFD. ChIP of DNMT1, DNMT3a, DNMT3b (**a**) and MBD2 (**b**) binding at *Ankrd26* promoter region (−553 bp/−348 bp). (**c**) MNase for Nuc-2 (−257 bp/−198 bp) and Nuc-1 (−84 bp/−25 bp) occupancy at *Ankrd26* promoter. (**d**) ChIP for acetyl-H4 enrichment at Nuc-2 and Nuc-1. (**e**) ChIP of RNA Pol II binding at *Ankrd26* TSS (+16 bp/+159 bp). (**a**,**b**) and (**d**,**e**), ChIP enrichment is relative to Input chromatin. (**a**–**e**), results are mean ± SD from three independent experiments. **p < 0.01 and ***p < 0.001 *vs* STD.

The molecular events upstream and downstream methylation-induced displacement of p300 binding to the *Ankrd26* promoter were subsequently analyzed. ChIP analysis revealed that binding of DNMT3a and DNMT3b, but not of DNMT1, to the *Ankrd26* promoter was increased in HFD-fed mice compared to controls (Fig. 4a). Interestingly, HFD increased the binding of methylation-dependent transcriptional repressor MBD2 as well (Fig. 4b)^23^.

### HFD affects histone acetylation, nucleosome positioning and RNA Pol II binding at the *Ankrd26* promoter in mice

Further analysis of the *Ankrd26* promoter using the NuPoP software predicted 2 nucleosomes (Nuc), Nuc-2 (−288 bp/−132 bp) and Nuc-1 (−105 bp/ + 41 bp), positioned between the p300 consensus sequence and the *Ankrd26* TSS. Micrococcal Nuclease (MNase) treatment of eAT chromatin from HFD- and STD-fed mice followed by qPCR revealed that HFD rendered the *Ankrd26* promoter less sensitive to nuclease digestion, increasing Nuc-2 and Nuc-1 positioning (Fig. 4c). Consistently, ChIP analysis showed that HFD feeding significantly lowered histone H4 acetylation at both nucleosomes in the HFD- compared to STD-fed mice (*p* < 0.001; Fig. 4d). The RNA Pol II binding to the *Ankrd26* TSS was also significantly lower in HFD-fed mice (*p* < 0.001; Fig. 4e). All together, these data indicate that methylation at the CpGs −436 and −431 bp and the subsequent p300 displacement from this region silenced *Ankrd26* expression through nucleosome remodeling at the *Ankrd26* promoter.

### *Ankrd26* silencing promotes secretion of pro-inflammatory chemokines by cultured adipocytes

**Figure 5:**
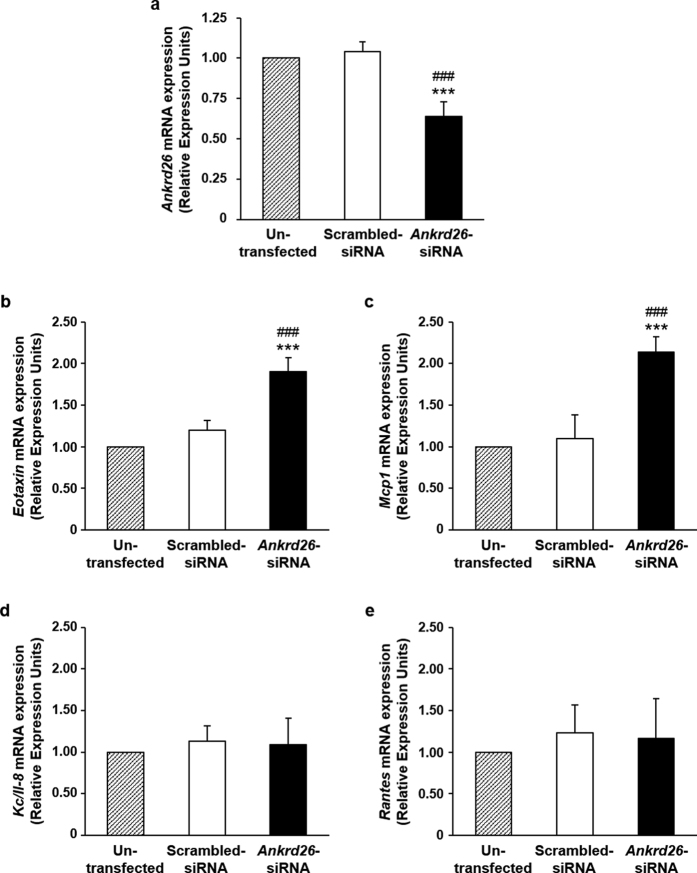
*Ankrd26* mRNA expression in *Ankrd26*-silenced mature adipocytes. 3T3-L1 mature adipocytes were silenced with 25 nmol/l of scrambled-siRNA or *Ankrd26*-siRNA for 48 h. Un-transfected cells were used to exclude transfection interference on mRNA expression. *Ankrd26* (**a**), *Eotaxin* (**b**), *Mcp1* (**c**), *Kc/Il-8* (**d**) and *Rantes* (**e**) mRNA levels were evaluated at the end of the experiment and expressed in Relative Expression Units (REU). Data are mean ± SD of determinations from three independent experiments. ***p < 0.001, *vs* Un-trasfected; ^###^*p* < 0.001, *vs* Scrambled-siRNA.

To assess the functional consequences of the HFD-induced epigenetic silencing of the *Ankrd26* gene, *Ankrd26* mRNA was reduced by about 35% by transfecting *Ankrd26*-specific siRNA in 3T3-L1 adipocytes (Fig. 5a). Silenced adipocytes showed enhanced secretion of the pro-inflammatory chemokines, Keratinocyte*-*derived Cytokine/Interleukine 8 (KC/IL-8), Eotaxin, Monocyte chemotactic protein 1 (MCP1) and Rantes (Table 2). These changes were accompanied by increased mRNA levels of *Eotaxin* and *Mcp1* with no change in *Kc/Il-8* and *Rantes* mRNAs (Fig. 5b,c,d and e). It appeared therefore that Ankrd26 physiologically controls the adipocyte pro-inflammatory secretion profile through effects occurring at different levels.

### *ANKRD26* expression negatively correlates with Body Mass Index (BMI) and inflammation markers in humans

**Figure 6:**
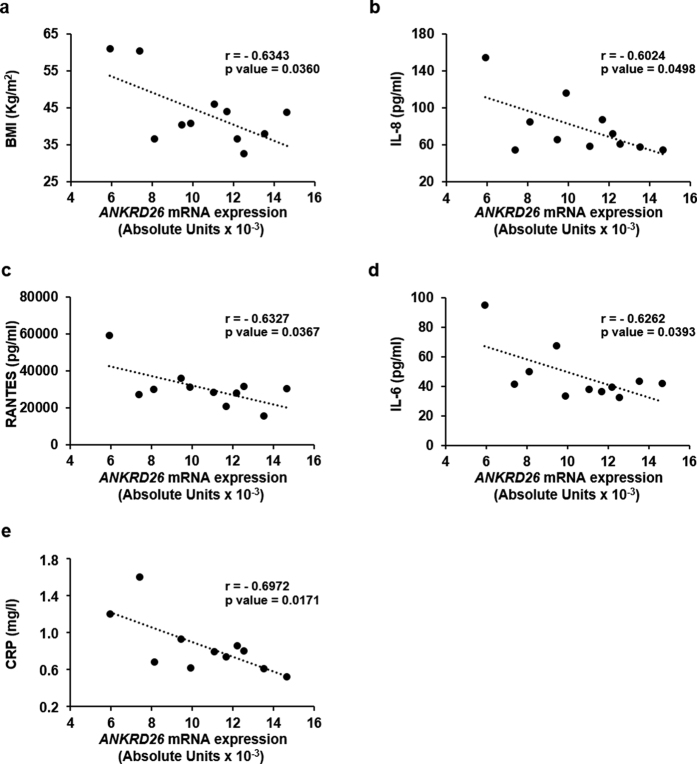
Associations between VAT *ANKRD26* mRNA expression, BMI and systemic inflammatory parameters in humans. Correlations of the human VAT *ANKRD26* mRNA expression with BMI (**a**) and with serum inflammatory markers, IL-8 (**b**), RANTES (**c**), IL-6 (**d**) and CRP (**e**) in normal glucose tolerant obese individuals (n = 11; 5 M/6 F). *r*, Pearson’s coefficient; *p* < 0.05 were considered statistically significant.

mRNA expression of *ANKRD26* in VAT was further examined in human obese subjects (Supplementary Table S1) in relation to BMI and inflammatory parameters. Interestingly, in obese subjects with normal glucose tolerance (NGT), *ANKRD26* expression in VAT was found to negatively correlate with BMI (Fig. 6a), with serum levels of the pro-inflammatory chemokines, IL-8 and RANTES and with serum levels of the inflammatory markers, IL-6 and C-reactive protein (CRP) (Fig. 6b,c,d and e). Altogether, these data indicate that, in obese humans, the reduction of *ANKRD26* gene expression is associated with increased body weight and with a pro-inflammatory status.

## Discussion

Epigenetic modifications represent a common mechanism through which both genetic and environmental exposures impact on the susceptibility to obesity and T2D^26^,^27^,^28^. Recent evidence has underlined the potential importance of epigenetic regulation of gene expression and function in obesity^26^. Methylation changes at the promoter of several genes have been identified in both human and rodent obesity^29^. Additionally, the exposure to high calorie diets, which promotes DNMT expression and enzymatic activities, impacts on DNA methylation profiles both at specific genes and at genome-wide level^22^,^30^, suggesting that DNA methylation changes play a role in the responses to fat and high-calorie diets^31^.

Our study revealed HFD-induced methylation of the *Ankrd26* promoter. We have no clue at the moment on the detailed mechanisms causing the increased methylation of *Ankrd26* in HFD-fed mice, but our results indicate that the HFD-induced hyper-methylation at *Ankrd26* promoter was concomitant with enhanced binding of *de novo* DNMT3a and DNMT3b to the same *Ankrd26* promoter region. These changes were followed by down-regulation of *Ankrd26* expression in the eAT. The epigenetic silencing of *Ankrd26* gene in eAT appear to depend, at least in part, on saturated fats, abundant in the HFD. Indeed, we found increased promoter DNA methylation and down-regulation of *Ankrd26* in 3T3-L1 adipocytes upon exposure to palmitate, a major component of the HFD, but not to oleate. At the variance with palmitate, cell exposure to leptin, whose serum concentration increases in relation to obesity^22^, showed no effect on *Ankrd26* expression and methylation. These findings suggest that specific nutritional components of the HFD may contribute to the epigenetic silencing of *Ankrd26* gene.

The HFD-induced changes in DNA methylation at *Ankrd26* promoter and gene expression result from a long-term diet exposure. Indeed, cytosine hyper-methylation at the *Ankrd26* promoter and gene silencing appeared in eAT from obese mice after a prolonged HFD feeding, while no evident difference was observed at earliest time-point. This time effect was associated with the eAT compensatory remodeling occurring in response to HFD. Indeed, eAT, along with other VAT depots, contributes to the inflammatory and metabolic complications in murine obesity^32^, and responds to HFD through different time-dependent changes^33^,^34^. Early upon HFD exposure (8–12 weeks), eAT expansion is accompanied by a major increase in adipocyte size. Upon more prolonged HFD exposure (20 weeks), however, eAT expansion is mainly sustained by increased adipogenesis and accompanied by enhanced secretion of inflammatory mediators, including Tumor necrosis factor alpha (TNF-α), IL-6, MCP1 and Rantes^33^,^34^,^35^. The mechanisms triggering this compensatory response in eAT have not been clarified yet but the present work now shows that they may involve HFD-induced *Ankrd26* down-regulation. Along with its role in feeding behavior and body fat accumulation^13^,^14^,^15^, *Ankrd26* has been identified as a regulator of adipogenesis *in vitro*^17^,^18^. Firstly, adipogenesis of 3T3-L1 cells is enhanced by selective silencing of the *Ankrd26* gene with an *Ankrd26*-specific shRNA^18^. Secondly, Mouse Embryonic Fibroblasts from *Ankrd26* mutant mice (MEFs *Ankrd26*^−/−^) have a higher rate of adipocyte differentiation. Indeed, the mRNA expression of the master regulator genes of differentiation process, *CCAAT enhancer-binding protein α (C/ebpα*), and *Peroxisome proliferator-activated receptor γ (Pparγ*), are up-regulated in MEFs *Ankrd26*^−/−^, indicating that this gene is involved in regulating both the pre-adipocyte commitment and differentiation^17^.

In this work, we further demonstrated enhanced expression and/or secretion of the pro-inflammatory chemokines Eotaxin, MCP1, KC/IL-8, and Rantes by 3T3-L1 adipocytes whose *Ankrd26* expression was silenced to levels similar to those occurring in response to HFD. These cytokines have been reported to contribute to adipose tissue inflammation^36^,^37^. Since secretion of Eotaxin, MCP1, KC/IL-8, and Rantes by the eAT also increases upon prolonged exposure to HFD^33^,^34^, our findings suggest the involvement of *Ankrd26* down-regulation in raising and/or sustaining the low-grade inflammatory response which occursin the eAT after long-termHFD feeding and is implicated in the development of insulin resistance and T2D^33^,^34^,^35^. This might represent a mechanism by which environmental cues are integrated at specific genomic loci, contributing to the metabolic disorder. The relevance of these observations to humans is supported by our further findings in obese individuals with normal glucose tolerance, revealing that the reduction of *ANKRD26* expression in VAT negatively correlates with the serum concentrations of inflammatory markers and pro-inflammatory chemokines, which are associated to obesity in humans^38^,^39^ and whose increased levels predict occurrence of T2D^38^,^39^,^40^,^41^,^42^,^43^. Cardamone *et al*. have recently shown in the adipose tissue the relevance of the cytosolic function of the Ankrd26 partner GPS2 (G protein pathway suppressor 2) to the prevention of uncontrolled activation of inflammatory programs^44^. Even though this issue deserves further mechanistic investigation, we suggest that Ankrd26 might work as a molecular regulator of inflammatory signaling pathways, at least in part, by facilitating the cytoplasmic localization of its interacting partner GPS2^18^. Therefore, *ANKRD26* down-regulation might represent an early event triggering chronic low-grade inflammatory response in human adipose tissue.

Detailed methylation analysis of the *Ankrd26* promoter showed that HFD induced specific methylations at −436 and −431 bp CpGs, thereby exerting a suppressive effect on *Ankrd26* promoter activity. Similar to DNA methylation, mutagenesis at the *Ankrd26* promoter showed that introduction of C → T mutation at −436 or −431 bp CpGs significantly reduces *Ankrd26* promoter activity. These results provide evidence that *i.*, these cytosine residues have functional significance to the *Ankrd26* gene expression; and *ii.*, the DNA methylation at these specific CpGs plays a functional role in the epigenetic repression of *Ankrd26* gene.

Current evidence supports a role for epigenetic changes in the regulation of metabolic diseases and in some cases, as in our study, it has been demonstrated that small methylation changes are associated with gene expression variability with significant effects on the phenotype^45^,^46^,^47^,^48^. In support of this concept, Barrès *et al*.^46^ have recently shown that hyper-methylation of the *Peroxisome proliferator-activated receptor γ coativator 1 α (PGC1α*) promoter modulates *PGC1α* expression, implying a mechanism for decreased mitochondrial content in skeletal muscle from T2D patients. Also, using a gene reporter assay, these same authors have demonstrated that the *in vitro* methylation of a single cytosine residue at the *PGC1α* promoter is responsible for the reduction of gene activity^46^.

CpG methylation generally affects transcription directly, by blocking the binding of transcriptional activators^24^,^49^, or indirectly, by recruiting DNA-binding proteins and co-repressor complexes that occupy the methylated promoters and facilitate the formation of heterochromatin^50^. In this study, we have further demonstrated that *i., in vitro*, the histone acetyltransferase/transcriptional coactivator p300 directly binds the consensus sequence at the *Ankrd26* promoter, containing the methylation sensitive cytosines −436 and −431 bp; *ii.*, hyper-methylation of these sites affects p300 binding and activity *in vivo* and *in vitro.* p300 regulates gene expression by acetylating both histones and transcriptional factors and plays a key role in modulating chromatin structure and function^25^,^26^. In this paper, we have also reported that HFD reduces histone H4 acetylation, increases nucleosome occupancy at the *Ankrd26* promoter, and impairs RNA Pol II binding to the *Ankrd26* TSS, suggesting that the HFD-dependent p300 displacement from the *Ankrd26* promoter silences *Ankrd26* gene. These findings are consistent with recent studies demonstrating that CpG methylation suppresses transcription of several genes by direct inhibition of p300 binding to their promoter sequences^51^,^52^,^53^. In conjunction with the inhibition of p300 binding, HFD induced the binding of MBD2 to the *Ankrd26* promoter in mice. MBD2 is a methyl-CpG binding protein and causes gene silencing by recruiting histone deacetylase at the methylated promoter regions^31^,^50^. Accordingly, we propose that the specific CpG methylation at the *Ankrd26* promoter leads to HFD-induced epigenetic gene silencing by triggering a cascade of events which involves DNA-associated regulatory proteins, such as p300 and MBD2, and changes in chromatin structure.

The potential relevance to humans of the findings reported in the present work is supported by our further evidence that VAT *ANKRD26* mRNA levels were negatively correlated with BMI in humans. Consistent with our results, very recent computational data from a genome-wide DNA methylation analysis in human adipose tissue have revealed that *ANKRD26* DNA methylation and mRNA expression correlate with BMI^53^. Thus, epigenetic regulation of *ANKRD26* gene may occur in humans as well.

In conclusion, our work reveals that the methylation of specific CpGs at the *Ankrd26* promoter occurs in mice during HFD treatment and causes the down-regulation of *Ankrd26* expression, at least in part, by impairing p300 binding to its promoter. We propose that the epigenetic silencing of the *Ankrd26* gene contributes to VAT inflammation following unhealthy dieting.

## Methods

### Animals, diets and tests

Animal experiments were performed in accordance with the Guide for the Care and Use of Laboratory Animals published by the National Institutes of Health (publication no. 85–23, revised 1996). Protocols were approved by the ethics committee of the “Federico II” University of Naples. Six-week-old C57BL/6 J male mice (n = 48) from Charles River Laboratories International, Inc. (Wilmington, MA) were housed in a temperature-controlled (22 °C) room with a 12 h light/dark cycle. Two weeks after arrival, mice were randomly divided into two groups of 12 mice each and were fed either a HFD (60 kcal% fat content; Research Diets formulas D12331; Research Diets, Inc., New Brunswick, NJ) or a standard chow diet (STD; 11 kcal% fat content; Research Diets formulas D12329; Research Diets, Inc.) for 8 and 22 weeks. The composition of these diets is reported in Supplementary Table S2. Body weight was recorded weekly throughout the study. The glucose tolerance test (GTT) and insulin tolerance test (ITT) were performed as described^14^,^16^. Blood glucose levels were measured using a glucometer (One Touch Lifescan, Milan, Italy). Mice were killed by cervical dislocation. eAT was collected from each mouse, snap frozen in liquid nitrogen and stored at −80 °C.

### Quantitative real-time PCR (qPCR) and western blot analysis

Tissues were homogenized by TissueLyser LT (Qiagen, Hilden, Germany) following manufacturer’s protocol. RNA and DNA were isolated using AllPrep DNA/RNA/miRNA Universal kit (Qiagen). cDNA synthesis and qPCR were performed as described^19^,^54^. Immunoblotting was carried out as indicated^14^. Antibodies against ANKRD26 (#SC-82505, Santa Cruz Biotechnology, Inc., Dallas, TX), and α-Tubulin (#MA1-19162, Sigma-Aldrich, St. Louis, MO) were used for protein detection.

### Methylated DNA Immuoprecipitation (MeDIP)

MeDIP assay was performed as described^19^. DNA methylation enrichment was evaluated on genomic DNA isolated from eAT of STD- and HFD-fed mice and from 3T3-L1 adipocytes. Sonicated pooled genomic DNA from eAT or cells was immuno-precipitated using anti-5meCpG (#ab10805, Abcam, Cambridge, MA) or mouse IgG (#I8765, Sigma-Aldrich) with anti-mouse IgG beads (Life Technologies, Carlsbad, CA). DNA methylation enrichment on recovered DNA was evaluated by qPCR. Samples were normalized to their respective input using the 2^−ΔCT^ method.

### Bisulfite sequencing

For bisulfite sequencing analysis, we used genomic DNA isolated from eAT of STD- and HFD-fed mice. Bisulfite conversion of DNA was performed with the EZ DNA Methylation Kit (Zymo Research, Orange, CA), following manufacturer’s instructions. Converted DNA was amplified by PCR. PCR products were cloned into the pGEM T-Easy vector (Promega, Madison, WI) and 10 clones for sample were sequenced on AB 3500 genetic analyzer (Life Technologies). DNA methylation percentage at the −436 and −431 bp CpGs for each mouse was calculated using the formula: DNA methylation % = [methylated CpGs/(methylated CpGs + unmethylated CpGs)]*100.

### Cloning strategy, site-direct mutagenesis and *in vitro* methylation

*Ankrd26* promoter (−733 bp/−344 bp) was amplified by PCR. The purified PCR fragment was cloned into the firefly luciferase reporter pCpGfree-promoter-Lucia vector (Invivogen, Toulouse, France). The following site-specific mutated constructs were generated by PCR-based mutagenesis: pCpG-*Ankrd26*-436, pCpG-*Ankrd26*-431, pCpG-*Ankrd26*-391. The wilde type (Wt) pCpG-*Ankrd26* vector, used as template, was removed from the PCR reaction by *DpnI* digestion (New England BioLabs, Ipswich, MA). Wt and mutated (mut) vectors were amplified into *E. coli* GT115 cells (Invivogen). Site-specific mutagenesis of each construct was validated by sequencing. *In vitro* methylation was performed using the *M.SsI* CpG methyltransferase following manufacturer’s protocol (New England BioLabs). Un-methylated DNA was obtained in the absence of *M.SsI*. Methylation was confirmed by resistance to *HpyCH4IV* digestion (New England BioLabs).

### Luciferase assay

NIH-3T3 cells were transfected with methylated or un-methylated Wt or mutagenized pCpG-*Ankrd26* vector and Renilla control vector (Promega) by lipofectamine (Life Technologies), following manufacturer’s instructions. Where indicated, cells were co-transfected with pCl.*p300* expression vector (Promega). Firefly luciferase activity of each transfection was normalized for transfection efficiency against Renilla luciferase activity.

### Chromatin Immunoprecipitation (ChIP) and Micrococcal Nuclease (MNase) assays

ChIP and MNase assays were performed as described^55^,^56^. Briefly, 100 mg of eAT were cross-linked with 1% formaldehyde for 15 min at 37 °C. For ChIP assay, sonicated chromatin was immuno-precipitated with the following antibodies: anti-p300 (#SC-585, Santa Cruz Biotechnology), anti-Ac-H4-K16 (#07-329, Millipore, Temecula, CA), anti-DNMT1 (#NB100-56519) and anti-DNMT3b (#NB300-516) from Novus Biologicals (Littleton, CO), anti-DNMT3a (#ab2850), anti-MBD2 (#ab38646), and anti-RNA Pol II (#ab5408) from Abcam and anti-rabbit IgG (#I8140) and anti-mouse IgG (#I8765) from Sigma-Aldrich. For MNase assay, nuclei were isolated from 100 mg of eAT, suspended in wash buffer (100 mmol/L Tris-HCl, 15 mmol/L NaCl, 60 mmol/L KCl, 1 mmol/L CaCl_2_) and treated with 200 U of MNase for 20 min at 37 °C. Cross-link reversal was performed at 65 °C for at least 16 h followed by an RNase and subsequent proteinase K digestion. DNA was purified by phenol–chloroform. Samples were then run on 1% agarose gel and the resulting mononucleosomal DNA fragments (~150 bp) were gel purified. For both assays, relative protein binding and nucleosome occupancy to the *Ankrd26* gene were evaluated on recovered DNA by qPCR. Samples were normalized to their respective input using the 2^−ΔCT^ method.

### Electrophoretic mobility shift assay (EMSA)

Protein-DNA complexes were detected using unlabeled or biotin end-labeled double-stranded DNA probes by annealing complementary oligonucleotides. Biotin 3′-end oligonucleotides, spanning the *Ankrd26* promoter sequence from −455 bp to −425 bp relative to the *Ankrd26* TSS, were from Sigma-Aldrich and, where indicated, were synthesized to incorporate methylated cytosines (^me^C). Binding reactions, consisting of biotin-labeled probe and NE, were performed using the LightShift kit (Thermo Fisher Scientific, Waltham, MA) following manufacturer’s instructions. Biotin-labeled probe (20 fmol) was added and the reaction was allowed to incubate for 20 min at room temperature. In the competition experiments, the nuclear extracts were preincubated with 200 molar excess of unlabeled probes for 20 min on ice. In super-shift experiments, 2 μg of p300 antibody (#SC-585, Santa Cruz Biotechnology) or 2 μg of rabbit IgG (#I8140, Sigma-Aldrich) was preincubated with nuclear extracts for 60 min on ice. Protein-DNA complexes were separated on native polyacrylamide gel, transferred onto nylon membrane and detected by the LightShift Chemiluminescent EMSA kit (Thermo Fisher Scientific) following manufacturer’s procedure.

### Primer Sequences

The list of oligonucleotides used for PCR, qPCR, MeDIP, bisulfite sequencing, ChIP, MNase, EMSA can be found as Supplementary Table S3.

### Fatty Acid/BSA complex solution preparation

Palmitate and oleate have been conjugated to fatty acid-free BSA (2:1 molar ratio Fatty Acid/BSA) as described in ref. 57. Briefly, a stock solution of palmitate (100 nM) was dissolved at 70 °C in 50% ethanol in a shaking water bath. In parallel, a fatty acid-free BSA solution was prepared at 55 °C in NaCl in a shaking water bath. Finally, the palmitate and the fatty acid-free BSA solutions were complexed at 55 °C in a shacking water bath, cooled to room temperature and sterile filtered. Oleate was complexed to the fatty acid-free BSA solutions following the same protocol. For fatty acid cell treatment, control adipocytes were treated with diluent only, corresponding concentrations of BSA and ethanol.

### Cell culture and transfection

3T3-L1 cells were grown and allowed to differentiate in mature adipocytes as described^19^. Mature adipocytes were *i.* silenced with 25 nmol/l of scrambled-siRNA or *Ankrd26*-siRNA for 48 h, or *ii.* treated with palmitate (0.250 mM; Sigma-Aldrich), or oleate (0.250 mM; Sigma-Aldrich) or corresponding vehicle (diluent solution with the same concentrations of BSA and ethanol of the Fatty Acid/BSA complex solution) for 96 h, or *iii.* treated with leptin (100 nM; R&D Systems, Minneapolis, CDN) or corresponding vehicle (20 mM Tris-HCl, pH 8.0) for 24 h. Adipokines were assayed in media from silenced cells by Bio*-*Plex Pro Mouse Cytokine Immunoassay following the manufacturer’s protocol (Bio-Rad, Hercules, CA). *Ankrd26* promoter methylation and gene expression were analyzed as previously described in this section.

### Patient enrollment and tests

Abdominal VAT biopsies and serum samples were from patients undergoing bariatric surgery. Eleven normal glucose tolerance (NGT) obese subjects were selected. Population characteristics are in Supplementary Table S1. Participants with metabolic and endocrine disorders, inflammatory diseases, previous or current malignancies, and/or treated with drugs able to interfere with the epigenome were excluded. Secreted mediators were assayed in serum samples by Bioplex multiplex Human Cytokine, Chemokine and Growth factor kit (Bio-Rad) following manufacturer’s protocol. *ANKRD26* gene expression was analyzed in VAT as previously described in this section.

### Ethics statement

This study adhered to the Declaration of Helsinki and has been reviewed and approved by the Ethics Committee of the “Federico II” University of Naples (Ethics Approval Number: No. 225_2013). Informed consent was obtained from all of enrolled individuals.

### Statistical procedures

The area under the curve (AUC) was calculated using the trapezoidal rule. Data are expressed as mean ± SD. Comparison between groups were performed using Student’s t-test or the one-way analysis of variance, as appropriate, using GraphPad Software (version 6.00 for Windows, La Jolla, CA). Correlation between two variables was calculated using the parametric Pearson r-test. *p* < 0.05 was considered statistically significant.

## Additional Information

**How to cite this article:** Raciti, G. A. *et al*. Specific CpG hyper-methylation leads to *Ankrd26* gene down-regulation in white adipose tissue of a mouse model of diet-induced obesity. *Sci. Rep.* **7**, 43526; doi: 10.1038/srep43526 (2017).

**Publisher's note:** Springer Nature remains neutral with regard to jurisdictional claims in published maps and institutional affiliations.

## Supplementary Material

Supplementary Information

## Figures and Tables

**Table 1:** Metabolic characteristics of HFD- and STD-fed mice.

Variable	16 week-old		30 week-old	
	STD (n = 12)	HFD (n = 12)	STD (n = 12)	HFD (n = 12)
Body weight (g)	24.7 ± 2.1	34.4 ± 3.2^a^	26.4 ± 2.8	38.8 ± 3.3^b,c^
Fasting glucose (mmol/l)	5.9 ± 0.9	7.7 ± 1.7^a^	5.8 ± 1.2	9.1 ± 1.8^b^
GTT AUC (mmol/l 120 min^−1^)	725.7 ± 103.3	1290.7 ± 162.4^a^	653.6 ± 150.0	1344.2 ± 172.2^b^
ITT AUCi (mmol/l 120 min^−1^)	741.6 ± 127.0	355.7 ± 132.2^a^	681.0 ± 128.9	312.9 ± 117.6^b^

8-week-old male C57BL/6 J mice were fed a high-fat diet (HFD) or a standard chow diet (STD) for 8 and 22 weeks. Body weight, fasting blood glucose, glucose tolerance test (GTT) Area Under the Curve (AUC) and insulin tolerance test (ITT) AUCi were reported. Data are mean ± SD of determinations. ^a^*p* < 0.001, 16-week-old HFD *vs* 16-week-old STD; ^b^*p* < 0.001, 30-week-old HFD *vs* 30-week-old STD; and ^c^*p* < 0.001, 30-week-old HFD *vs* 16-week-old HFD.

**Table 2:** Effect of *Ankrd26* gene silencing on adipocyte-released chemokines/cytokines.

Variable	3T3-L1 Adipocytes		
	Un-transfected	Scrambled-siRNA	*Ankrd26*-siRNA
Eotaxin (pg/ml)	514.34 ± 73.25	521.16 ± 82.90	705.92 ± 98.99^b,d^
G-CSF (pg/ml)	6.45 ± 0.96	7.11 ± 0.22	7.14 ± 0.90
IL-4 (pg/ml)	3.41 ± 0.32	3.21 ± 0.44	3.81 ± 0.51
IL-5 (pg/ml)	0.88 ± 0.14	0.79 ± 0.16	0.83 ± 0.33
KC/IL-8 (pg/ml)	565.39 ± 15.32	588.67 ± 30.86	702.58 ± 34.08^a,c^
IL-17 (pg/ml)	1.44 ± 0.62	1.72 ± 0.51	1.56 ± 0.42
MCP1 (pg/ml)	1758.04 ± 72.31	1718.77 ± 248.29	2500.31 ± 225.38^b,d^
MIP1β (pg/ml)	1.32 ± 0.75	1.14 ± 0.52	1.17 ± 0.37
Rantes (pg/ml)	27.95 ± 2.74	33.31 ± 8.88	53.45 ± 2.35^b,d^
TNFα (pg/ml)	4.98 ± 1.46	5.29 ± 1.31	5.57 ± 1.81

3T3-L1 mature adipocytes were silenced with 25 nmol/l of scrambled-siRNA or *Ankrd26*-siRNA for 48 h. Conditioned media were collected for 24 h in Dulbecco’s modified Eagle’s medium without serum and with 0.5% BSA. Adipokines were then assayed using the Bio*-*Plex Pro Mouse Cytokine Immunoassay. Un-transfected cells were also used to exclude transfection interference on adipokine secretion. Detectable adipokines are reported. Data are mean ± SD of determinations from three independent experiments. ^a^*p* < 0.001 and ^b^*p* < 0.01, *vs* Un-transfected; ^c^*p* < 0.001 and ^d^*p* < 0.01, *vs* Scrambled-siRNA. Granulocyte-colony stimulating factor, G-CSF; Interleukin, IL; Keratinocyte*-*derived Cytokine/Interleukine 8, KC/IL-8; Monocyte chemotactic protein 1, MCP1; Macrophage inflammatory protein 1 beta, MIP1β; Tumor necrosis factor alpha, TNFα.

## References

[b1] Eds Cavan D., da Rocha Fernandes J., Makaroff L., Ogurtsova K. & Webber S. Brussels, Belgium: International Diabetes Federation. International Diabetes Federation. IDF Diabetes Atlas 2015, 7th edition (pdf available online) Executive summary, 12–19 (Last date of access: 29/11/2016) http://www.diabetesatlas.org (2015).

[b2] Hu F. B. Globalization of diabetes: the role of diet, lifestyle, and genes. Diabetes Care 34, 1249–1257 (2011). 21617109 10.2337/dc11-0442 PMC3114340

[b3] Ng M. . Global, regional, and national prevalence of overweight and obesity in children and adults during 1980–2013: a systematic analysis for the Global Burden of Disease Study 2013. Lancet 384, 766–781 (2014). 24880830 10.1016/S0140-6736(14)60460-8 PMC4624264

[b4] Seki Y., Williams L., Vuguin P. M. & Charron M. J. Minireview: Epigenetic programming of diabetes and obesity: animal models. Endocrinology 153, 1031–1038 (2012). 22253432 10.1210/en.2011-1805 PMC3281534

[b5] Rakyan V. K., Down T. A., Balding D. J. & Beck S. Epigenome-wide association studies for common human diseases. Nat. Rev. Genet. 12, 529–541 (2011). 21747404 10.1038/nrg3000 PMC3508712

[b6] Toperoff G. . Genome-wide survey reveals predisposing diabetes type 2-related DNA methylation variations in human peripheral blood. Hum. Mol. Genet. 21, 371–383 (2012). 21994764 10.1093/hmg/ddr472 PMC3276288

[b7] Bell C. G. . Integrated genetic and epigenetic analysis identifies haplotype-specific methylation in the FTO type 2 diabetes and obesity susceptibility locus. PLoS One 5, e14040 (2010). 21124985 10.1371/journal.pone.0014040 PMC2987816

[b8] Virtue S. & Vidal-Puig A. Adipose tissue expandability, lipotoxicity and the Metabolic Syndrome–an allostatic perspective. Biochim. Biophys. Acta, 1801, 338–349 (2010). 20056169 10.1016/j.bbalip.2009.12.006

[b9] Neeland I. J. . Dysfunctional adiposity and the risk of prediabetes and type 2 diabetes in obese adults. JAMA 308, 1150–1159 (2012). 22990274 10.1001/2012.jama.11132 PMC3556508

[b10] Hayashi T. . Visceral adiposity is an independent predictor of incident hypertension in Japanese Americans. Ann. Intern. Med. 140, 992–1000 (2004). 15197016 10.7326/0003-4819-140-12-200406150-00008

[b11] Boyko E. J., Fujimoto W. Y., Leonetti D. L. & Newell-Morris L. Visceral adiposity and risk of type 2 diabetes: a prospective study among Japanese Americans. Diabetes Care 23, 465–471 (2000). 10857936 10.2337/diacare.23.4.465

[b12] Hayashi T. . Visceral adiposity and the risk of impaired glucose tolerance: a prospective study among Japanese Americans. Diabetes Care 26, 650–655 (2003). 12610016 10.2337/diacare.26.3.650

[b13] Bera T. K. . A model for obesity and gigantism due to disruption of the Ankrd26 gene. Proc. Natl. Acad. Sci. USA 105, 270–275 (2008). 18162531 10.1073/pnas.0710978105 PMC2224199

[b14] Raciti G. A., Bera T. K., Gavrilova O. & Pastan I. Partial inactivation of Ankrd26 causes diabetes with enhanced insulin responsiveness of adipose tissue in mice. Diabetologia 54, 2911–2922 (2011). 21842266 10.1007/s00125-011-2263-9 PMC3881194

[b15] Acs P. . A novel form of ciliopathy underlies hyperphagia and obesity in Ankrd26 knockout mice. Brain Struct. Funct. 220, 1511–1528 (2015). 24633808 10.1007/s00429-014-0741-9 PMC4601608

[b16] Dong C. . Possible genomic imprinting of three human obesity-related genetic loci. Am. J. Hum. Genet. 76, 427–437 (2005). 15647995 10.1086/428438 PMC1196395

[b17] Fei Z., Bera T. K., Liu X., Xiang L. & Pastan I. Ankrd26 gene disruption enhances adipogenesis of mouse embryonic fibroblasts. J. Biol. Chem. 286, 27761–27768 (2011). 21669876 10.1074/jbc.M111.248435 PMC3149366

[b18] Liu X. F. . ANKRD26 and its interacting partners TRIO, GPS2, HMMR and DIPA regulate adipogenesis in 3T3-L1 cells. PLoS One 7, e38130 (2012). 22666460 10.1371/journal.pone.0038130 PMC3364200

[b19] Parrillo L. . Hoxa5 undergoes dynamic DNA methylation and transcriptional repression in the adipose tissue of mice exposed to high-fat diet. Int. J. Obes. (Lond) 40, 929–37 (2016). 26980478 10.1038/ijo.2016.36

[b20] Gesta S. . Evidence for a role of developmental genes in the origin of obesity and body fat distribution. Proc. Natl. Acad. Sci. USA 103, 6676–6681 (2006). 10.1073/pnas.0601752103 PMC1458940 16617105

[b21] Sackmann-Sala L., Berryman D. E., Munn R. D., Lubbers E. R. & Kopchick J. J. Heterogeneity among white adipose tissue depots in male C57BL/6J mice. Obesity 20, 101–111 (2012). 21779095 10.1038/oby.2011.235 PMC3666351

[b22] Shen W. . Epigenetic modification of the leptin promoter in diet-induced obese mice and the effects of N-3 polyunsaturated fatty acids. Sci. Rep. 4, 5282 (2014). 24923522 10.1038/srep05282 PMC5381469

[b23] Bird A. DNA methylation patterns and epigenetic memory. Genes Dev. 16, 6–21 (2002). 11782440 10.1101/gad.947102

[b24] Ito T., Ikehara T., Nakagawa T., Kraus W. L. & Muramatsu M. p300-mediated acetylation facilitates the transfer of histone H2A-H2B dimers from nucleosomes to a histone chaperone. Genes. Dev. 14, 1899–1907 (2000). 10921904 PMC316828

[b25] Liu X. . The structural basis of protein acetylation by the p300/CBP transcriptional coactivator. Nature 451, 846–850 (2008). 18273021 10.1038/nature06546

[b26] Drong A. W., Lindgren C. M. & McCarthy M. I. The genetic and epigenetic basis of type 2 diabetes and obesity. Clin. Pharmacol. Ther. 92, 707–715 (2012). 23047653 10.1038/clpt.2012.149 PMC7116747

[b27] Raciti G. A. . Understanding type 2 diabetes: from genetics to epigenetics. Acta Diabetol. 52, 821–827 (2015). 25841587 10.1007/s00592-015-0741-0

[b28] Raciti G. A. . Personalized medicine and type 2 diabetes: lesson from epigenetics. Epigenomics 6, 229–238 (2014). 24811791 10.2217/epi.14.10

[b29] Desiderio A. . Epigenetics: spotlight on Type 2 diabetes and obesity. J. Endocrinol. Invest. 10.1007/s40618-016-0473-1 (2016). 27180180

[b30] van Dijk S. J., Molloy P. L., Varinli H., Morrison J. L. & Muhlhausler B. S. Members of EpiSCOPE. Epigenetics and human obesity. Int. J. Obes. 39, 85–97 (2015). 10.1038/ijo.2014.34 24566855

[b31] Voisin S. . Dietary fat quality impacts genome-wide DNA methylation patterns in a cross-sectional study of Greek preadolescents. Eur. J. Hum. Genet. 23, 654–662 (2015). 25074463 10.1038/ejhg.2014.139 PMC4402618

[b32] Berry D. C., Stenesen D., Zeve D. & Graff J. M. The developmental origins of adipose tissue. Development 140, 3939–3949 (2013). 24046315 10.1242/dev.080549 PMC3775412

[b33] Strissel K. J. . Adipocyte death, adipose tissue remodeling, and obesity complications. Diabetes 56, 2910–2918 (2007). 17848624 10.2337/db07-0767

[b34] Wang Q. A., Tao C., Gupta R. K. & Scherer P. E. Tracking adipogenesis during white adipose tissue development, expansion and regeneration. Nat. Med. 19, 1338–1344 (2013). 23995282 10.1038/nm.3324 PMC4075943

[b35] Strissel K. J. . T-cell recruitment and Th1 polarization in adipose tissue during diet-induced obesity in C57BL/6 mice. Obesity 18, 1918–1925 (2010). 20111012 10.1038/oby.2010.1 PMC2894258

[b36] Kim H. J. . Expression of eotaxin in 3T3-L1 adipocytes and the effects of weight loss in high-fat diet induced obese mice. Nutr. Res. Pract. 5, 11–19 (2011). 21487491 10.4162/nrp.2011.5.1.11 PMC3061264

[b37] Huber J. . CC chemokine and CC chemokine receptor profiles in visceral and subcutaneous adipose tissue are altered in human obesity. J. Clin. Endocrinol. Metab. 93, 3215–3221 (2008). 18492752 10.1210/jc.2007-2630

[b38] Stępień M. . Obesity indices and inflammatory markers in obese non-diabetic normo- and hypertensive patients: a comparative pilot study. Lipids Health Dis. 13, 29 (2014). 24507240 10.1186/1476-511X-13-29 PMC3921991

[b39] Dandona P., Aljada A. & Bandyopadhyay A. Inflammation: the link between insulin resistance, obesity and diabetes. Trends Immunol. 25, 4–7 (2004). 14698276 10.1016/j.it.2003.10.013

[b40] Barzilay J. I. . The relation of markers of inflammation to the development of glucose disorders in the elderly: the Cardiovascular Health Study. Diabetes. 50, 2384–92001 (2001). 11574423 10.2337/diabetes.50.10.2384

[b41] Pradhan A. D. . C-reactive protein, interleukin 6, and risk of developing type 2 Diabetes mellitus. JAMA. 286, 327–34 (2001). 11466099 10.1001/jama.286.3.327

[b42] Herder C. . Association of systemic chemokine concentrations with impaired glucose tolerance and type 2 diabetes: results from the Cooperative Health Research in the Region of Augsburg Survey S4 (KORA S4). Diabetes. 54, Suppl 2 S11–7 (2005). 16306328 10.2337/diabetes.54.suppl_2.s11

[b43] Herder C. . Immunological and cardiometabolic risk factors in the prediction of type 2 diabetes and coronary events: MONICA/KORA Augsburg case-cohort study. PLoS One. 6, e19852 (2011). 21674000 10.1371/journal.pone.0019852 PMC3108947

[b44] Cardamone M. D. . A protective strategy against hyperinflammatory responses requiring the non-transcriptional actions of GPS2. Mol. Cell. 46, 91–104 (2012). 22424771 10.1016/j.molcel.2012.01.025 PMC3327812

[b45] Gracia A. . Fatty acid synthase methylation levels in adipose tissue: effects of an obesogenic diet and phenol compounds. Genes Nutr. 9, 411 (2014). 24903834 10.1007/s12263-014-0411-9 PMC4169062

[b46] Barrès R. . Non-CpG methylation of the PGC-1alpha promoter through DNMT3B controls mitochondrial density. Cell Metab. 10, 189–198 (2009). 19723495 10.1016/j.cmet.2009.07.011

[b47] Barrès R. . Weight loss after gastric bypass surgery in human obesity remodels promoter methylation. Cell Rep. 3, 1020–1027 (2013). 23583180 10.1016/j.celrep.2013.03.018

[b48] Paternain L. . Transcriptomic and epigenetic changes in the hypothalamus are involved in an increased susceptibility to a high-fat-sucrose diet in prenatally stressed female rats. Neuroendocrinology 96, 249–260 (2012). 22986707 10.1159/000341684

[b49] Kuroda A. . Insulin gene expression is regulated by DNA methylation. PLoS One 4, e6953 (2009). 19742322 10.1371/journal.pone.0006953 PMC2735004

[b50] Ballestar E. & Wolffe A. P. Methyl-CpG-binding proteins. Targeting specific gene repression. Eur. J. Biochem. 268, 1–6 (2001). 11121095 10.1046/j.1432-1327.2001.01869.x

[b51] Li H. P. . Aberrantly hypermethylated Homeobox A2 derepresses metalloproteinase-9 through TBP and promotes invasion in Nasopharyngeal carcinoma. Oncotarget 4, 2154–2165 (2013). 24243817 10.18632/oncotarget.1367 PMC3875777

[b52] Rönn T. . Impact of age, BMI and HbA1c levels on the genome-wide DNA methylation and mRNA expression patterns in human adipose tissue and identification of epigenetic biomarkers in blood. Hum Mol Genet 24, 3792–3813 (2015). 25861810 10.1093/hmg/ddv124

[b53] Raciti G. A. . Glucosamine-induced endoplasmic reticulum stress affects GLUT4 expression via activating transcription factor 6 in rat and human skeletal muscle cells. Diabetologia 53, 955–965 (2010). 20165829 10.1007/s00125-010-1676-1

[b54] Haim Y., Tarnovscki T., Bashari D. & Rudich A. A chromatin immunoprecipitation (ChIP) protocol for use in whole human adipose tissue. Am. J. Physiol. Endocrinol. Metab. 305, E1172–1177 (2013). 24002573 10.1152/ajpendo.00598.2012

[b55] Carey M. & Smale S. T. Micrococcal Nuclease-Southern Blot Assay: I. MNase and Restriction Digestions. CSH Protoc. pdb.prot4890 (2007). 10.1101/pdb.prot4890 21356981

[b56] Spector A. A. Fatty acid binding to plasma albumin. J. Lipid. Res. 16, 165–179 (1975). 236351

[b57] Kuehnen P. . An Alu element-associated hypermethylation variant of the POMC gene is associated with childhood obesity. PLoS Genet. 8, e1002543 (2012). 22438814 10.1371/journal.pgen.1002543 PMC3305357

