# Supplementary material for: Specific CpG hyper-methylation leads to Ankrd26 gene down-regulation in white adipose tissue of a mouse model of diet-induced obesity
Source: Sci Rep. 2017 Mar 7;7:43526. doi: 10.1038/srep43526 (PMC5339897; doi:10.1038/srep43526)
Supplement: Supplementary Information [file srep43526-s1.pdf]

Supplementary Information

Specific CpG hyper-methylation leads to *Ankrd26* gene down-regulation in white adipose tissue of a mouse model of diet-induced obesity

Gregory A. Raciti[1,2 §], Rosa Spinelli[1,2 §], Antonella Desiderio[1,2], Michele Longo[1,2], Luca Parrillo[1,2], Cecilia Nigro[1,2], Vittoria D'Esposito[1,2], Paola Mirra[1,2], Francesca Fiory[1,2], Vincenzo Pilone[3], Pietro Forestieri[4], Pietro Formisano[1,2], Ira Pastan[5], Claudia Miele[1,2 *] and Francesco Beguinot[1,2 *]

[1] URT of the Institute of Experimental Endocrinology and Oncology "G. Salvatore", National Council of Research, Naples, 80131, Italy.

[2] Department of Translational Medical Sciences, University of Naples "Federico II", Naples, 80131, Italy.

[3] Bariatric and Metabolic Surgery Unit, University of Salerno, Salerno, 84084, Italy.

[4] Department of Clinical Medicine and Surgery, University of Naples "Federico II", Naples, 80131, Italy.

[5] Laboratory of Molecular Biology (LMB), National Cancer Institute (NCI), National Institute of Health (NIH), Bethesda, MD 20892, USA.

[§] These Authors contributed equally to this work.

[*] Corresponding Authors: Claudia Miele and Francesco Beguinot, URT of the Institute of Experimental Endocrinology and Oncology "G. Salvatore", National Council of Research, Naples, Italy & Department of Translational Medical Sciences, University of Naples "Federico II", Naples, Via Pansini 5, 80131, Italy, Phone: +39 081 7463248, Fax: +39 081 7463235. E-mail: c.miele@ieos.cnr.it (C.M.); beguino@unina.it (F.B.).

Supplementary Figures

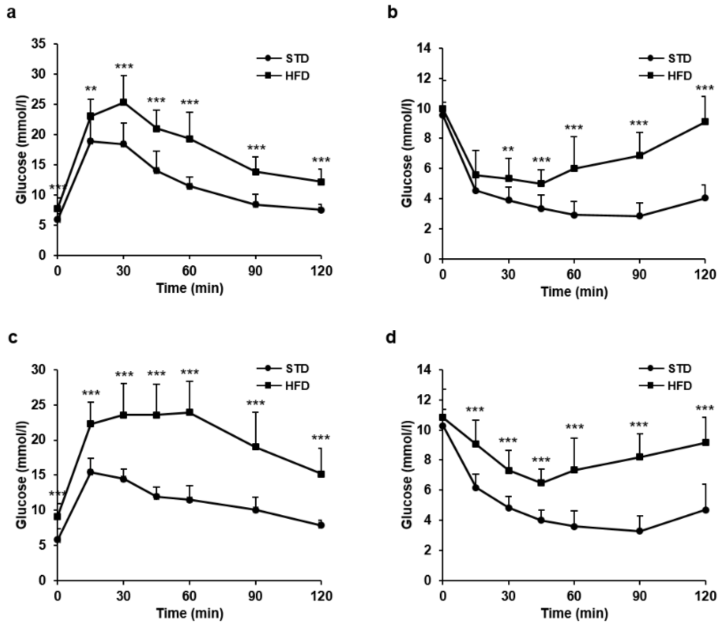

Supplementary Figure 1

Supplementary Figure 1. Glucose Tolerance Test (GTT) and Insulin Tolerance Test (iTT) in HFD- and STD-fed mice. 8-week-old male C57BL/6J were fed a high-fat diet (HFD) or a standard chow diet (STD) for 8 and 22 weeks. (**a, c**) HFD-fed (black squares/lines) and STD-fed (black circles/lines) mice were subjected to GTT upon (**a**) 8 weeks, and (**c**) 22 weeks of diet regimens. Mice were fasted for 16 h and subjected to i.p. glucose loading (2 g/kg body weight). Blood glucose levels were determined at various times. (**b, d**) Mice as above underwent ITT upon (**b**) 8 weeks, and (**d**) 22 weeks of diet regimens. Random fed mice were injected i.p. with insulin (0.75 U/kg body weight), followed by determination of blood glucose levels at the indicated times. Blood glucose levels were determined at various times. For experiments (**a-d**), values are mean ± SD of determinations in twelve mice per group. $^{**}p<0.01$, and $^{***}p<0.001$ *vs* STD.

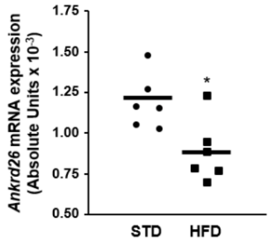

Supplementary Figure 2

Supplementary Figure 2. *Ankrd26* expression in mesenteric VAT upon 22 weeks of HFD feeding. qPCR of *Ankrd26* mRNA for HFD- (n=6) and STD-fed (n=6) mice. mRNA levels are expressed in absolute units (AU). *$p<0.05$ *vs* STD.

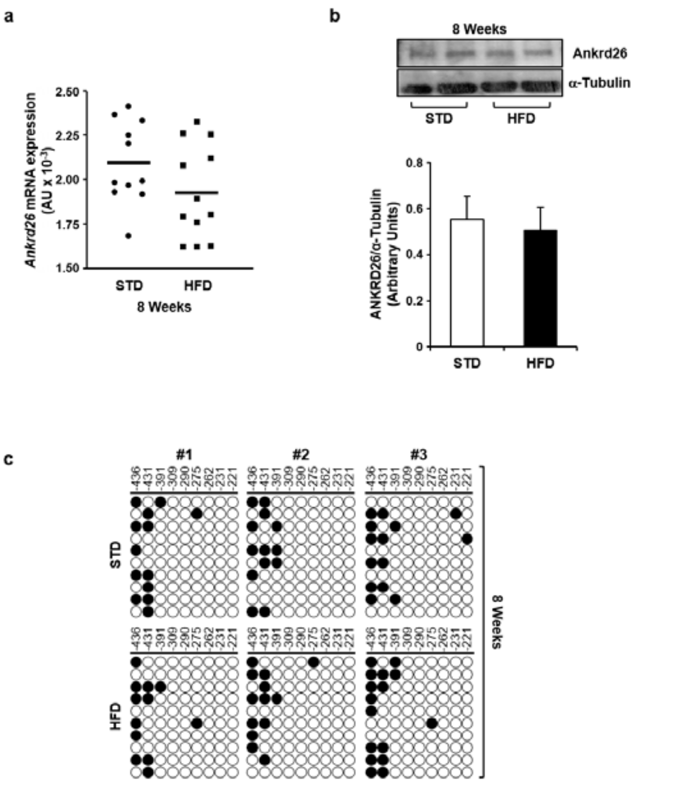

Supplementary Figure 3

Supplementary Figure 3. Ankrd26 expression and DNA methylation in eAT upon 8 weeks of HFD feeding. (**a**) qPCR of *Ankrd26* mRNA for HFD- (n=12) and STD-fed (n=12) mice. mRNA levels are expressed in absolute units (AU). (**b**) Representative western blot for ANKRD26 and α-Tubulin. Results are means ± SD from three independent experiments. Uncut western blot images are in the Supplementary Figure S5. (**c**) Bisulfite sequencing of *Ankrd26* promoter region (-436 bp/-221 bp) in HFD- (n=3) and STD-fed (n=3) mice. Each row indicates sequencing results of ten independent clones. White circles, un-methylated CpGs; black circles, methylated CpGs. CpG position relative to *Ankrd26* TSS is shown above each column.

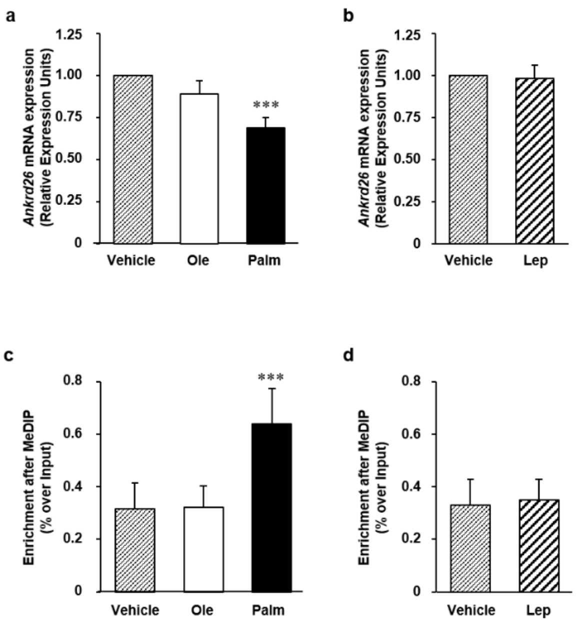

Supplementary Figure 4

Supplementary Figure 4. *Ankrd26* expression and DNA methylation changes in mature adipocytes upon treatment with palmitate (Palm), or oleate (Ole) for 96 h, or leptin (Lep) for 24 h. 3T3-L1 mature adipocytes were treated with palmitate (0.250 mM; Palm), or oleate (0.250 mM; Ole) or corresponding vehicle (diluent solution with the same concentrations of BSA and ethanol of the Fatty Acid/BSA complex solution; Vehicle) for 96 h, or treated with leptin (100 nM; Lep) or corresponding vehicle (20 mM Tris-HCl, pH 8.0; Vehicle) for 24 h. (**a**, **b**) qPCR of *Ankrd26* mRNA. (**c, d**) MeDIP-qPCR of segment 1 (S1; -462 bp/-193 bp) of *Ankrd26* promoter region. **a**-**d**, results are mean ± SD from three independent experiments. ***p<0.001 *vs* Vehicle.

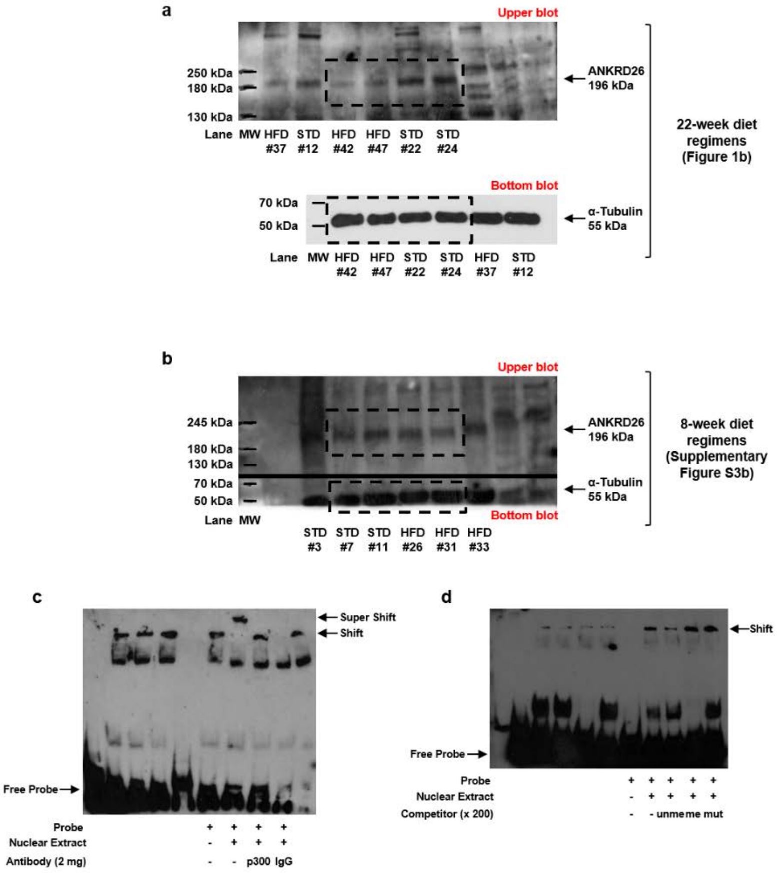

Supplementary Figure 5

Supplementary Figure 5. Full length blots and gels. (**a-b**) eAT proteins (50µg) were separated by SDS-PAGE, blotted on PVDF membranes and probed with antibodies against ANKRD26 and α-Tubulin as described in Method section. (**a**) Ankrd26 expression in eAT from mice fed HFD or STD for 22 weeks. Representative uncut western blot images for ANKRD26 (upper blot) and α-Tubulin (bottom blot). Upper blot: lanes 2, 4 and 5, proteins from mice fed HFD for 22 weeks; lanes 3, 6 and 7, proteins from mice fed STD for 22 weeks; and Lane 1, protein markers. Bottom blot: lanes 2, 3 and 6, proteins from mice fed HFD for 22 weeks; lanes 4, 5 and 7, proteins from mice fed STD for 22 weeks; and Lane 1, protein markers. Samples in the upper and bottom blots are from the same experiments and blots have been processed in parallel. (**b**) Ankrd26 expression in eAT from mice fed HFD or STD for 8 weeks. Representative uncut western blot images for ANKRD26 (upper blot) and α-Tubulin (bottom blot); the black line delineates the boundary between the blots. Lanes 6, 7 and 8, proteins from mice fed HFD for 8 weeks. Lanes 3, 4 and 5, proteins from mice fed STD for 8 weeks. Lane 1, protein markers. (**c-d**) Protein/double-stranded biotinylated *Ankrd26* probe complexes were separated on native polyacrylamide gel, transferred onto nylon membrane and detected by the LightShift Chemiluminescent EMSA kit, as described in Method section. (**c**) Representative EMSA super-shift assay with an anti-p300 antibody (lane 8) or a rabbit IgG (lane 9). (**d**) Representative EMSA competition assay with 200-fold molar excess of un-labeled un-methylated (unme; lane 8), methylated (me; lane 9) or mutagenized (mut; lane 10) competitor.

Supplementary Tables

Supplementary Table S1. Baseline characteristics of the obese subjects with normal glucose tolerance

	Subjects, n=11(5M/6F)	
	Mean ± SD	Range
**General variables**		
Age (years)	27.4 ± 5.6	19.0 - 46.0
BMI (Kg/m^2)	43.6 ± 9.3	32.6 - 61.0
Fasting glucose (mmol/l)	4.8 ± 0.4	4.3 - 5.8
Fasting insulin (pmol/l)	105.9 ± 31.5	63.2 -154.9
HOMA-IR	3.0 ± 1.2	1.3 - 5.1
OGTT plasma glucose (mmol/l)	6.4 ± 0.8	5.0 - 7.0

Data are means ± SD of determinations. For each variable, the lowest and the higher values are shown. BMI, Body Mass Index; OGTT, Oral Glucose Tolerance Test.

Supplementary Table S2. Composition of diets, D12329 and D12331

Formula	D12329		D12331	
	g%	kcal%	g%	kcal%
Protein	16.8	16.4	23	16.4
Carbohydrate	74.3	73.1	35.5	25.5
Fat	4.8	10.5	35.8	58

Formula	D12329		D12331	
Ingredient	g	kcal	g	kcal
Casein, 30 Mesh	228	912	228	912
DL-Methionine	2	0	2	0
Maltodextrin 10	170	680	170	680
Corn Starch	0	0	0	0
Sucrose	835	3340	175	700
Soybean Oil	25	225	25	225
Coconut Oil, Hydrogenated	40	360	333.5	3001.5
Mineral Mix S10001	40	0	40	0
Sodium Bicarbonate	10.5	0	10.5	0
Potassium Citrate, 1 H_2O	4	0	4	0
Choline Bitartrate	2	0	2	0
FD&C Blue Dye	0.1	0	0.1	0
Vitamin Mix V10001	10	40	10	40
Total	1366.6	5557	1000.1	5558.5

Composition of Vitamin Mix V10001

Vitamin Mix V10001	g	Amount in 10 g
Vitamin A	0.8	4000 IU
Vitamin D3	1000 IU	100,000 IU/gm
Vitamin E	10 50 IU	500 IU/gm
Menadion	0.08	0.5 mg
Biotin	2	0.2 mg
Vitamin B12	1	10 µg
Folic Acid	0.2	2 mg
Niacin	3	30 mg
Pantothenic Acid	1.6	16 mg
Vitamin B6	0.7	7 mg
Vitamin B2	0.6	6 mg
Vitamin B1	0.6	6 mg

For further details, please refer to http://www.researchdiets.com/opensorcediets/stock-diets/surwit-diets.

Supplementary Table S3. Primer sequences

**Technique**	**Gene/Region**	**Primer sequence**
**qPCR**	*Ankrd26*	F 5’-CTTTGGACGCGAGAGTGCTA-3’<br>R 5’-AGCAGTCCTGTCCTTCTTGTC-3’
	β-Actin	F 5’-AAGATCAAGATCATTGCTCCTCCTG-3’<br>R 5’-AGCTCAGTAACAGTCCGCCT-3’
	ANKRD26	F 5’-GTATGCTAGTAGTGGTCCTGC-3’<br>R 5’-GTAGGCCTTCCTTCATCCTCAT-3’
	RPL13A	F 5’-CTTTCCGCTCGGCTGTTTTC-3’<br>R 5’-GCCTTACGTCTGCGGATCTT-3’
	Eotaxin	F 5’-AGTCGGGAGAGCCTACAGAG-3’<br>R 5’-AAGTTGGGATGGAGCCTGG-3’
	Mcp1	F 5’-CTGTAGTTTTTGTCACCAAGCTCA-3’<br>R 5’-GTGCTGAAGACCTTAGGGCA-3’
	Kc/Il-8	F 5’-TAGGCATCTTCGTCCGTCCC-3’<br>R 5’-CCTTCACCCATGGAGCATCA-3’
	Rantes	F 5’-AGGAACCGCCAAGTGTGTGCC-3’<br>R 5’-AGTGGCATCCCCAAGCTGGC-3'
**MeDIP-qPCR**	*Ankrd26* S1	F 5’-CTGCAAGGCTTCAACAGGAA-3’<br>R 5’-ACAAAATCTCTTCCCTTACTCTTCC-3’
	Ankrd26 S2	F 5’-TGGAAACAACACACTGCCCA-3’<br>R 5’-AACGCAGCTAGGGCACTTAT-3’
**Bisulfite sequencing**	*Ankrd26* Region 1	F 5'-TAAATTATTTAGTTTAATAAAATTTTTTTT-3'<br>R 5'-TCTTTACTATTCAAAAAATCAAAAC-3'
	Ankrd26 Region 2	F 5'-TTTTGATTTTTTGAATAGTAAAGAAGG-3'<br>R 5'-CCTTATAAATCTTACCCATATCCTTATC-3'
**Cloning and mutagenesis**	*Ankrd26* Wild Type	F 5’-GCCCTAGGCCTTTGAGGTGAGTTGTGGCT-3’<br>R 5’- GCGGATCCGGCAGTTAAACCTGTTGGGG-3’
	Ankrd26 Mut -436	F 5’-TCCCTTACTCTTCCA**T**GCCACGATTCCATCC-3’<br>R 5’- GGATGGAATCGTGGC**A**TGGAAGAGTAAGGGA-3’
	Ankrd26 Mut -431	F 5’- ACTCTTCCACGCCA**T**GATTCCATCCATGC-3’<br>R 5’- GCATGGATGGAATC**A**TGGCGTGGAAGAGT-3’
	Ankrd26 Mut -391	F 5’- GTAGTTTACTTTTTGATTAT**T**GCCTACCTAGAGTCCAATAG-3’<br>R 5’- CTATTGGACTCTAGGTAGGC**A**ATAATCAAAAAGTAAACTAC-3’
**ChIP-qPCR and MNase**	p300/DNMTs/MBD2<br>bs *Ankrd26*	F 5’-ACCTCCCATCAGCTTGTCTAAC-3’<br>R 5’-GCCTTGTGTTTCAGTGGCAG-3’
	-257/-198<br>Nuc-2 *Ankrd26*	F 5’-TCCTGGAGTACTGGGTCTGG-3’<br>R 5’-AGGCTTCAACAGGAATGGGG-3’
	-84/-25<br>Nuc-1 *Ankrd26*	F 5’-CCTGAACAGCAAAGAAGGCG-3’<br>R 5’-CAGCGACCAGTGCCCAG-3’
	+16/+159<br>TSS *Ankrd26*	F 5’-GTTGCTAGTTTGCAGCTCGG-3’<br>R 5’-CAACGTTGGCTGACAACACA-3’

**EMSA**	Labeled *Ankrd26* probe	F 5'- CTCTTCCCTTACTCTTCCACGCCACGATTCC-3'[Btn]<br>R 5'- GGAATCGTGGCGTGGAAGAGTAAGGGAAGAG-3'[Btn]
	Un-labeled *Ankrd26* probe	F 5'- CTCTTCCCTTACTCTTCCACGCCACGATTCC -3'<br>R 5'- GGAATCGTGGCGTGGAAGAGTAAGGGAAGAG-3'
	Un-labeled Methy *Ankrd26* probe	F 5'- CTCTTCCCTTACTCTTCCA^{me}CGCCA^{me}CGATTCC-3'<br>R 5'- GGAAT^{me}CGTGG^{me}CGTGGAAGAGTAAGGGAAGAG -3'
	Un-labeled Mut *Ankrd26* probe	F 5'- CTCTTCCCTTACTCTTCCA**T**GCCA**T**GATTCC -3'<br>R 5'- GGAATC**A**TGGC**A**TGGAAGAGTAAGGGAAGAG -3'

Methylated CpGs are indicated as ^{me}C. Mutated cytosines (C → T) are indicated in bold. F, forward; R, reverse.
